# Temporal dynamics of attentional selection in adult male carriers of the fragile X premutation allele and adult controls

**DOI:** 10.3389/fnhum.2015.00037

**Published:** 2015-02-05

**Authors:** Ling M. Wong, Flora Tassone, Susan M. Rivera, Tony J. Simon

**Affiliations:** ^1^MIND Institute, University of California Davis School of MedicineSacramento, CA, USA; ^2^Department of Psychiatry and Behavioral Sciences, University of California Davis School of MedicineSacramento, CA, USA; ^3^Department of Biochemistry and Molecular Medicine, University of California Davis School of MedicineSacramento, CA, USA; ^4^Department of Psychology, University of California DavisDavis, CA, USA; ^5^Center for Mind and Brain, University of California DavisDavis, CA, USA

**Keywords:** fragile X, *FMR1* gene, attentional blink, attention, temporal processing, executive function, inhibition, letter number sequencing

## Abstract

Carriers of the fragile X premutation allele (fXPCs) have an expanded CGG trinucleotide repeat size within the *FMR1* gene and are at increased risk of developing fragile x-associated tremor/ataxia syndrome (FXTAS). Previous research has shown that male fXPCs with FXTAS exhibit cognitive decline, predominantly in executive functions such as inhibitory control and working memory. Recent evidence suggests fXPCs may also exhibit impairments in processing temporal information. The attentional blink (AB) task is often used to examine the dynamics of attentional selection, but disagreements exist as to whether the AB is due to excessive or insufficient attentional control. In this study, we used a variant of the AB task and neuropsychological testing to explore the dynamics of attentional selection, relate AB performance to attentional control, and determine whether fXPCs exhibited temporal and/or attentional control impairments. Participants were adult male fXPCs, aged 18–48 years and asymptomatic for FXTAS (*n* = 19) and age-matched male controls (*n* = 20). We found that fXPCs did not differ from controls in the AB task, indicating that the temporal dynamics of attentional selection were intact. However, they were impaired in the letter-number sequencing task, a test of executive working memory. In the combined fXPC and control group, letter-number sequencing performance correlated positively with AB magnitude. This finding supports models that posit the AB is due to excess attentional control. In our two-pronged analysis approach, in control participants we replicated a previously observed effect and demonstrated that it persists under more stringent theoretical constraints, and we enhance our understanding of fXPCs by demonstrating that at least some aspects of temporal processing may be spared.

## 1. Introduction

Fragile X premutation carriers (fXPCs) are defined as individuals who have a 55–200 CGG repeat expansion in the *FMR1* gene, which is located on the X chromosome. The premutation allele is so named because it can expand in subsequent generations into the full mutation allele (>200 CGG). Individuals with a full mutation often have Fragile X Syndrome (FXS), which is associated with a ~30% chance of developing autism (Rogers et al., 2001) and low levels of the *FMR1* protein (FMRP). The premutation allele has an estimated prevalence of 1 in 260– 813 in males and 1 in 113–259 in females (Hagerman, 2008). Male fXPCs have an elevated risk of developing fragile X-associated tremor/ataxia syndrome (FXTAS), a neurodegenerative disorder with age-dependent penetrance (17%, 38%, 47%, and 75% [lower-bound estimates] for participants aged 50–59, 60–69, 70–79, and ≥ 80 years, respectively), characterized by intention tremor, ataxia, and parkinsonism (Jacquemont et al., 2004). The FXTAS phenotype is thought to be due to a toxic gain of function of excess *FMR1* mRNA, which is associated with increasing CGG repeat length (Hagerman and Hagerman, 2004). This potential mechanism is supported by associations between CGG repeat length and age of onset of FXTAS (Tassone et al., 2007); level of motor impairment in fXPCs (Leehey et al., 2008); negative associations of CGG repeat length with brain volume, packing density of middle cerebellar peduncle, and gray matter density of the dorsomedial frontal lobes (Cohen et al., 2006; Hashimoto et al., 2011a,b); and negative associations of both CGG repeat length and *FMR1* mRNA with the connectivity strength of the superior cerebellar peduncle (Wang et al., 2013).

Increased CGG repeat length is associated with executive function impairment in fXPCs without FXTAS, so it is possible that subtle cognitive impairments precede motor impairments. Firstly, males with FXTAS can exhibit cognitive decline, particularly in executive functions such as inhibitory control and working memory (WM) (Grigsby et al., 2007, 2008; Brega et al., 2008; Cornish et al., 2009, 2011). Men with FXTAS have impairments in various aspects of inhibitory control, including interference control, cognitive control, and behavioral inhibition, as assessed by the Stroop Color-Word test, Behavioral Dyscontrol Scale, and Controlled Oral Word Association Test (Grigsby et al., 2006, 2007, 2008; Brega et al., 2008). Secondly, men with longer CGG repeat length are impaired in some of these tasks (Grigsby et al., 2006, 2007), and in male fXPCs asymptomatic for FXTAS, CGG repeat length modulates the effect of age on a behavioral inhibition task (Cornish et al., 2011; Hunter et al., 2012).

In addition to inhibitory control impairments, evidence suggests that processing of temporal information may be affected in fXPCs. This is suggested by relatively high FMRP in magnocellular (M) layers of the lateral geniculate nucleus (Zangenehpour et al., 2009). The M pathway of visual processing feeds into cortical areas responsible for motion perception and visuomotor coordination, which requires use of spatial and temporal information. Meanwhile, the parvocellular (P) pathway feeds into cortical regions responsible for color perception and object recognition, which relies much less heavily on spatial and temporal information (Van Essen and Gallant, 1994). As described in a recent review (Kraan et al., 2013), several studies report impaired visuospatial and temporal (e.g., ordering or memory) function in both male and female fXPCs. For example, fXPCs were found to exhibit a specific impairment in M pathway processing (Kéeri and Benedek, 2009, 2012), and functioning in this pathway has been linked to FMRP expression in males who were not fXPCs (Kéeri and Benedek, 2011). Female and male fXPCs exhibit impairments in tasks of visuospatial function (Goodrich-Hunsaker et al., 2011a,b; Hocking et al., 2012; Wong et al., 2012). In a mouse model of the premutation allele, female CGG knock-in (KI) mice demonstrated a CGG repeat length-sensitive impairment in temporal ordering (Hunsaker et al., 2010) and impaired temporal memory for spatial locations (Borthwell et al., 2012). To date, there has only been one study of temporal memory in human fXPCs. This study found that while typical adults show increased activation in the when pathway (i.e., right temporoparietal junction: TPJ) during temporal relative to spatial WM retrieval, adult fXPCs of both sexes failed to exhibit this pattern (Kim et al., 2014).

However, to date there have been no studies of temporal attention in fXPCs of any age. Due to the sparse literature in this area, it is unclear whether temporal processing and/or temporal attention are specifically impaired in fXPCs. For our purposes in this study, we define *temporal attention* as the temporal dynamics by which attention is deployed, and *temporal processing* as the low-level computation of temporal parameters of incoming stimuli (e.g., which item appeared first, next, or last).

The attentional blink (AB) task has been used to examine the temporal dynamics of attentional selection. During rapid serial visual presentation (RSVP) of stimuli, the “attentional blink” refers to a decrement in identification accuracy for the second of two targets in the stream, which occurs when the targets occur in close temporal proximity (Raymond et al., 1992). In a variation of the classic AB paradigm, the temporal distance between two targets remained constant, but the attend instructions and presence of intervening distractors were manipulated (Di Lollo et al., 2005; Dell'Acqua et al., 2009). In that variant, an AB was observed when distractors intervened between targets, but not when targets were continually presented (contiguous target condition). A trailing target presented in very close temporal proximity to the first target is often identified just as well as the first target, a phenomenon known as “Lag 1 sparing.” Thus, this lack of an AB for additional trailing targets has been referred to as “spreading the sparing” (Olivers et al., 2007).

This AB task provides an interesting paradigm from which to explore both the temporal dynamics of attentional selection and attentional control. Timing was held constant across conditions in the task variant used by Di Lollo et al. (2005). Thus, the authors proposed that the AB was due to a “temporary loss of attentional control” (TLC model), and not due to a purely temporal limitation of how quickly items can be processed and consolidated into WM. According to their model, RSVP processing is governed by an attention filter endogenously configured to select targets and exclude distractors, and by a central processor which switches between monitoring and consolidation processes. After the first target is identified, the central processor switches from monitoring incoming stimuli to consolidating the first target into memory. No longer under control of the central processor, the attention filter can be exogenously reconfigured by incoming stimuli. If targets continue to appear, the filter is already configured to select them, so targets proceed into consolidation. However, if a distractor appears, the filter is reconfigured, such that when a trailing target appears, the filter is no longer optimally configured for target selection, so processing of the trailing target suffers. While the TLC model posits that the AB is due to insufficient attentional control, alternate models posit the opposite, that the AB is due to excessive control (a more extensive description of models accounting for the AB is included in the Discussion). In such models, the appearance of a distractor can trigger a suppressive response, such that the trailing target is suppressed instead of selected (Raymond et al., 1992; Olivers et al., 2007; Taatgen et al., 2009). Therefore, the AB task can be modeled as assessing the temporal dynamics of attentional selection as well as inhibition of distracting information.

In this study, we had several aims. First, we sought to replicate and extend the “spreading the sparing” effect, previously observed in undergraduate students, in a sample of adults aged 18–48 years. We constrained analyses to trials in which preceding targets were accurately identified, or accurately identified and reported in the correct order, and discuss how results relate to predictions of competing models. Second, we sought to more fully characterize the temporal dynamics of attentional selection. We accomplished this by examining perception of temporal order and inter-target competition for attention resources. Third, we sought to determine whether the AB is better modeled as due to excessive or insufficient control. To do this, we related AB task performance to measures of executive WM. We will discuss how results from these three aims relate to existing models of attention in control participants. Our final aim was to determine whether fXPCs were impaired relative to controls on any of these measures, and whether performance was associated with CGG or *FMR1* mRNA. Understanding the core cognitive phenotype in fXPCs may facilitate early identification of individuals most at risk for developing FXTAS.

Inhibition of distracting information is a critical component of successful WM performance. Thus, this variant of the AB task, by manipulating attention demands (i.e., the type of items to be attended or ignored) and WM load (i.e., two or three items), allows for investigation into the nature of the interaction between selective attention and WM. Specifically, in Aim 1 we compare performance when three targets are presented vs. performance when there are only two targets but with an intervening distractor. We also contrast this to performance when one target is a member of the distractor stimulus set. We find evidence that when an item from the distractor set is a target and enters WM, selective attention to a subsequent target from the target set is impaired. We also examine the effect of increasingly stringent criteria on attention demands on WM performance. In Aim 2 we examine how two items in WM compete for resources. In Aim 3, we explore the relationship between attention and WM by examining whether two commonly used tests of executive WM predict the AB phenomenon. Together, these aims capitalize on the unique design of the AB task to better understand how selective attention and WM interact and relate to attentional control.

## 2. Materials and methods

### 2.1. Participants

Participants were 41 males aged 18–48 years, including 20 control participants and 21 fXPCs. FXPCs had at least one family member with FXS. All had normal, or corrected to normal, vision.

Participants were recruited through the NeuroTherapeutics Research Institute (NTRI) at the Medical Investigation of Neurodevelopmental Disorders (MIND) Institute at the University of California, Davis Medical Center, and from the community through recruitment advertisements. FXPCs were recruited from known FXS pedigrees, and controls were recruited from pedigrees or the community. Exclusion criteria were: acute medical condition such as renal, liver, cardiac, or other disease that may be associated with brain atrophy or dysfunction; current or past history of major DSM-IV Axis I psychiatric disorder; history of head trauma, toxic encephalopathy, encephalitis, or bacterial meningitis; history of alcoholism or drug problem; and use of current medications that affect cerebral blood flow (e.g., beta blockers). This study was approved by the Institutional Review Board of the University of California, Davis and conformed to institutional and federal guidelines for the protection of human participants. Written informed consent was obtained before participation from all participants.

### 2.2. Procedure

We conducted this experiment as part of a larger study. The study visit involved administration of cognitive tests and a blood draw. All fXPCs were evaluated by a physician and determined to be asymptomatic for FXTAS following published criteria (Jacquemont et al., 2003; Bacalman et al., 2006). All control participants completed the Tremor Disability Rating Scale (Jacquemont et al., 2004). Of 31 common actions, one control participant reported difficulty or disability on two actions (“using eyedrops” and “threading a needle”). This participant's performance was not extreme, so he was included in all analyses as a control participant.

#### 2.2.1. Molecular assays

Molecular data were *FMR1* CGG repeat length and mRNA expression level. Genomic DNA was isolated from peripheral blood leukocytes using standard methods (Puregene Kit; Gentra Inc., Valencia, CA, USA). Repeat length was determined using Southern blot analysis and PCR amplification of genomic DNA as described previously (Tassone et al., 2008). All quantifications of *FMR1* mRNA were performed using a 7900 Sequence detector (PE Biosystems).

#### 2.2.2. Psychological assessment

Full scale IQ (FSIQ) was measured using either the Wechsler Adult Intelligence Scale, third edition (WAIS-III) (Wechsler, 1997) or the Wechsler Abbreviated Scale of Intelligence (WASI) (Wechsler, 1999). Due to time constraints during testing, FSIQ data were not available from all participants.

***2.2.2.1. Working memory measures***. Two sub-scales from the FSIQ test were identified. Digit span backward total score is a measure of the ability to hold and process information in WM. In this task, participants hear a sequence of digits and must repeat them in the reverse order. Letter-number sequencing requires holding as well as manipulating information in WM. In this task, participants hear a mixed sequence of digits and letters, and must report the digits in ascending order, and the letters in alphabetical order. Thus, both tasks require storage and executive WM, while letter-number sequencing additionally requires the ability to switch attention between task sets. These measures were used to examine the effect of executive WM on task performance.

***2.2.2.2. ADHD assessment***. ADHD status was measured using the 66-item Conners' Adult ADHD Rating Scale (CAARS) (Conners et al., 1999). Participants completed a self-report, and an observer-report was completed by a spouse, partner, family member, or close friend. Scores were adjusted according to established age and sex norms. Sub-scores measured DSM-IV inattentive, hyperactive-impulsive, and total ADHD symptoms. Due to time constraints during testing and inability to collect observer reports during testing, ADHD data were not available from all participants.

#### 2.2.3. Temporal attention task

Stimuli were presented via E-Prime 2.0.8.90 (http://www.pstnet.com) on a Tobii T120 eye tracking system (http://www.tobii.com). Participants were seated 60 cm from the eye-tracking monitor in a chin rest to maintain head position. Participants performed practice trials and were observed during task performance to ensure appropriate task performance.

This task replicated parameters used previously (Di Lollo et al., 2005). Participants pressed a button to initiate each trial. A fixation cross appeared in the center of the screen for 500 ms, and was followed by a RSVP stream. The stream consisted of 5–10 digits followed by a 3-item target sequence, and ended with one digit which served as a perceptual mask. Length of the initial stream of digits (5–10 items) was pseudo-randomized so that every six trials, all possible trial lengths were presented. The target sequence consisted of three letters or a letter-number-letter sequence, depending on condition, which will be described shortly. All stimuli appeared in Century Gothic font and subtended approximately 1.45 ° in width and 2.8 ° in height. Stimuli were black characters on a gray background presented for 30 ms, with a blank inter-stimulus interval of 70 ms. Digits ranged from 0–9, and were presented in randomized order, with the constraint that no digit was identical to the previously presented digit. Letters comprised the English alphabet, excepting I, O, Q, or Z. Letters were not repeated within a trial, and were pseudo-randomized so that no sequences formed words or common abbreviations. Participants were informed of the excluded letters and that digits would range from 0–9.

**Figure 1:**
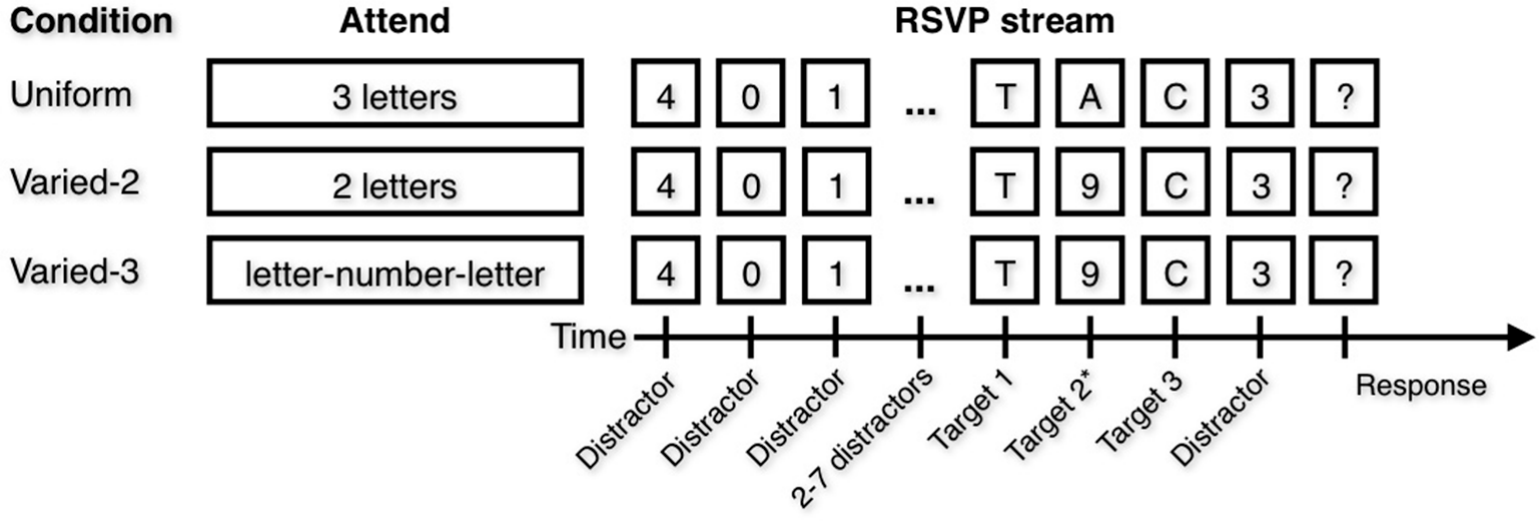
**Attentional Blink (AB) task**. In each blocked condition, participants were instructed to attend to certain types of items. Note that the Varied conditions differ only in the attend instruction. In the Varied–3 condition, the correct number is the number that occurs temporally between the two letters. Target position 2 is marked with an asterisk because in the Varied-2 condition, there are only two targets.

In each of three conditions (Figure 1), participants attended to 2–3 items (positions T1, T2, and T3) within the target sequence. In the Uniform condition, participants attended to three letters. They were informed they would view a stream of digits with three letters in the stream, and they were instructed to attend all three letters and to type them into the keyboard in any order when prompted by a question mark at the end of the trial. In the Varied–2 condition, participants attended to two letters but not to digits. Instructions were similar to those in the Uniform condition except participants were informed there would be two letters and they should attend both letters. In the Varied–3 condition, participants attended to two letters as well as the digit that appeared temporally between them. Instructions were similar to those in the Uniform condition except participants were informed there would be a letter-number-letter sequence embedded in the stream, and they were to identify both letters and the number that appeared in between the letters, but not any of the other numbers. The Varied–2 and Varied–3 conditions differed only in instruction; in both conditions, a digit was presented temporally between two letters. In other words, the same pattern of stimuli were presented in both conditions, but all digits were distractors to be ignored in the Varied–2 condition; meanwhile, in the Varied–3 condition the digit appearing between two letter targets was to be attended, and all remaining digits were distractors to be ignored. Each of the three conditions was presented as a block, with specific instructions preceding each block. Participants completed 10 practice trials and 60 experimental trials of each block. Trial stimuli were identical for all participants, although the order was randomized across participants. Block order was counterbalanced across participants (Uniform/Varied–2/Varied–3 and Varied–3/Varied–2/Uniform).

Thus, letters were always targets, and digits were only targets in the Varied-3 condition and if the digit occurred after the first letter and before the second letter. In the Uniform condition, the three letters were always presented in direct succession, while in the Varied-2 and Varied-3 condition, the first letter was always immediately followed by a digit, which was always immediately followed by the second letter. In these two conditions, only a single digit appeared between letters. In all conditions, the first target letter appeared in position 6–11 in the RSVP stream, and the second letter (in the Varied–2 and Varied–3 conditions) or last letter (in the Uniform condition) appeared in position 8–13 in the stream.

As described by Di Lollo et al. (2005), the dependent measure was target identification accuracy for each target in each condition. A decrement in T3 accuracy relative to T1 accuracy is defined as the AB. According to the TLC model, an AB in the Varied–2 condition would indicate that when a distractor appears between two successive targets, the distractor disrupts the selection filter. Thus, when the trailing target appears, the filter is not optimally configured to process that target. This non-optimal configuration results in poor identification accuracy for the trailing target. In the Uniform condition, no distractors appear between successive targets, so the selection filter remains optimally configured, and the trailing target does not suffer a decrement in identification accuracy.

The Varied–3 condition was included to address the concern that poor T3 performance in the Varied–2 condition might be due to the need to suppress the intervening distractor. Suppression processes might be interfering with target identification processes. Thus, in the Varied–3 condition, like in the Uniform condition, participants must report all three presented items (i.e., no suppression). Similar T1 and T3 performance between Varied–2 and Varied–3 conditions indicates that even when participants are told to ignore all digits, they perform as if they are attending to the intervening digit.

### 2.3. Statistical analyses

In Di Lollo et al. (2005), target identification accuracy was calculated regardless of accuracy for other targets within the trial (“non-conditional accuracy”). However, as Dell'Acqua et al. (2009) note, for proper interpretation of T2 accuracy, T1 responses must be accurate (“conditional accuracy”). This is an important requirement to interpret whether or not T2 accuracy decrements result from ongoing T1 processing. To replicate previous findings as well as extend their interpretation, we calculated both non-conditional and conditional accuracy.

Specifically, for Aim 1, three analyses of variance (ANOVAs) were run. In the first ANOVA, the dependent variable was target identification accuracy. For the second ANOVA, the data were divided such that T2 and T3 accuracy were computed only from trials in which the preceding target(s) were correctly identified (T1 and T2 in the Uniform and Varied–3 conditions; T1 in the Varied–2 condition). Thus, in the second ANOVA, the dependent variable was target identification accuracy with conditional accuracy. For the third ANOVA, the data were further divided such that T2 and T3 accuracy were computed only from trials in which the preceding target(s) were correctly identified and reported in the correct ordinal position. Thus, in the third ANOVA, the dependent variable was target identification accuracy with conditional accuracy and order. In each of the three ANOVAs, we examined main effects of Group, Condition, and Target; two-way interactions of Group X Condition, Group X Target, and Condition X Target; and the three-way interaction of Group X Condition X Target. The AB was defined as T1 accuracy minus T3 accuracy in a particular condition. The “T1 enhancement effect” was defined as higher T1 accuracy in the Varied–2 or Varied–3 condition than Uniform condition.

For Aim 2, we included only trials in which T1 and T3 were accurately identified, and if T2 was to be attended (i.e., Uniform and Varied–3 trials), if it was correctly identified. Thus, we included only trials in which all targets were correctly identified. The dependent variable was percentage of trials in which T1 and T3 were reported in the correct relative order (i.e., T1 reported before T3, even if T2 was also reported). We ran an ANOVA of Group X Condition on order accuracy. Ordinal position of T2 report was ignored to avoid potentially confounding effects on perception of temporal order. Specifically, because letters occurred only in the T1 and T3 positions, while both letters and numbers occurred at T2, a mismatch in stimulus set between T2 and flanking targets (i.e., in a letter-number-letter sequence), could lead to arbitrary differences in response order (e.g., letter-letter-number or number-letter-letter).

To examine the effect of inter-target competition, we tested whether (A) T1 accuracy predicted T3 accuracy, and (B) T3 accuracy predicted T1 accuracy. The Uniform and Varied–3 conditions have a memory load of three items, as opposed to two in the Varied–2 condition, so we excluded the Varied–2 condition from these analyses. We ran an ANOVA of (A) Group X Condition X T1 accuracy on T3 accuracy, and of (B) Group X Condition X T3 accuracy on T1 accuracy.

For Aim 3, the magnitude of the AB in the Varied–3 condition with conditional accuracy was calculated for each participant. We chose the Varied–3 condition, as opposed to the Varied–2 condition, because the instruction was to attend three contiguous items. Thus, while an AB should be observed in both the Varied–2 and Varied–3 conditions, the continuous attention required in the Varied–3 condition allows for ease of interpretation. To examine whether the AB relates to excessive or insufficient attentional control and whether this relationship differs between groups, we used linear regression to assess the effect of group on AB magnitude, the relationship between AB magnitude and neuropsychological measures of executive WM (i.e., digit span backward total score or letter-number sequencing score), and whether the relationship between neurospsychological measures and AB magnitude differed between groups. Specifically, we tested models with AB magnitude as the outcome variable and Group X digit span backward score as predictors or Group X letter-number sequencing score as predictors. To examine whether our results were due to differences in overall attention function, we examined the effect of ADHD status on task performance. Specifically, we used linear regression to examine the effect of group and either self- or observer-report of ADHD symptoms on AB magnitude.

FXTAS exhibits age-dependent penetrance and fXPCs are at elevated risk for developing FXTAS, so we reasoned that even though our sample was asymptomatic for FXTAS, we should test for effects of age. Accordingly, for each analysis we also ran models with an additional age covariate. For all analyses, the addition of age did not change the pattern of results. Therefore, we report results from analyses without age as a predictor. For all analyses, a *p* < 0.05 was considered statistically significant.

## 3. Results

### 3.1. Study sample

A total of 20 control participants and 21 fXPCs performed the tasks (Table 1). The mean age (± SD) was 29.95 (6.48) years for controls and 32.17 (7.74) for fXPCs, which did not differ significantly (*t* = −1.00, *p* = 0.32). The mean CGG repeat length was 29.40 (5.63) (range: 20–44) for controls and 97.33 (24.62) (range: 55–146) for fXPCs, which differed significantly (*t* = −12.20, *p* < 0.001). One participant expressed two variants of the premutation allele (120 and 156). His performance was not extreme in any task, so he was included in all analyses. To assess the effect of CGG repeat length on performance, separate correlations were tested using the mean (138) or higher (156) CGG value. The mean *FMR1* mRNA value was 1.41 (0.23) (range: 1.10–1.76) for controls and 3.05 (1.37) (range: 1.85–7.81) for fXPCs, which differed significantly (*t* = −5.09, *p* < 0.001). FSIQ data were missing from 4 control participants. Mean FSIQ was 119.40 (14.20) for controls, and 115.37 (13.66) for fXPCs, and did not differ between groups (*t* = 0.84, *p* = 0.41). ADHD self-report data were available from 19 controls and 18 fXPCs (i.e., missing from 1 control and 3 fXPCs), and observer-report data were available from 18 controls and 14 fXPCs (i.e., missing from 2 controls and 7 fXPCs).

**Table 1:** **Participant descriptive statistics and *FMR1* measures**.

	**Control**			**fXPC**			**Df**	***t***	***p*-value**
	***N***	**Range**	**Mean (SD)**	***N***	**Range**	**Mean (SD)**			
Age (yrs)	20	18–40	30.0 (6.48)	21	22–48	32.2 (7.74)	39	−1.00	0.32
Full scale IQ	16	87–142	120 (13.74)	21	91–143	117 (13.69)	35	0.64	0.53
*Digit span backward*		4–15	8.4 (2.92)		4–12	7.8 (2.28)	35	0.69	0.49
*Letter-number sequencing*		9–20	14.2 (3.12)		7–15	11.9 (2.30)	35	2.46	0.02
CGG repeats	15	20–44	29.40 (5.63)	21	55–146	97.33 (24.62)	34	−12.20	<0.001
mRNA	13	1.10–1.76	1.41 (0.23)	18	1.85–7.81	3.05 (1.37)	29	−5.09	<0.001

*A p < 0.05 was considered statistically significant*.

### 3.2. Overall task performance

All participants performed at > 50% accuracy for T1 identification in the Uniform condition (T1 Uniform accuracy). Thus, participants performed well above chance and were judged able to perform the task. To identify any outlier participants, we defined outliers as having T1 Uniform accuracy greater than 3 times the interquartile range (IQR) or less than 3 times the IQR within each group. No outlier participants were identified, so all participants were included in the analyses.

### 3.3. Replicating and extending the “spreading the sparing” effect

In Aim 1, we sought to replicate and extend the “spreading the sparing” effect, previously observed in undergraduate students, in a sample of adults aged 18–48 years (Di Lollo et al., 2005). To address this, we first replicated the method of analysis, and then constrained the data by requiring that preceding targets must be accurately identified (“conditional accuracy”), and then also requiring that targets must be reported in the correct order (“conditional accuracy + order”).

#### 3.3.1. Non-conditional accuracy

**Figure 2:**
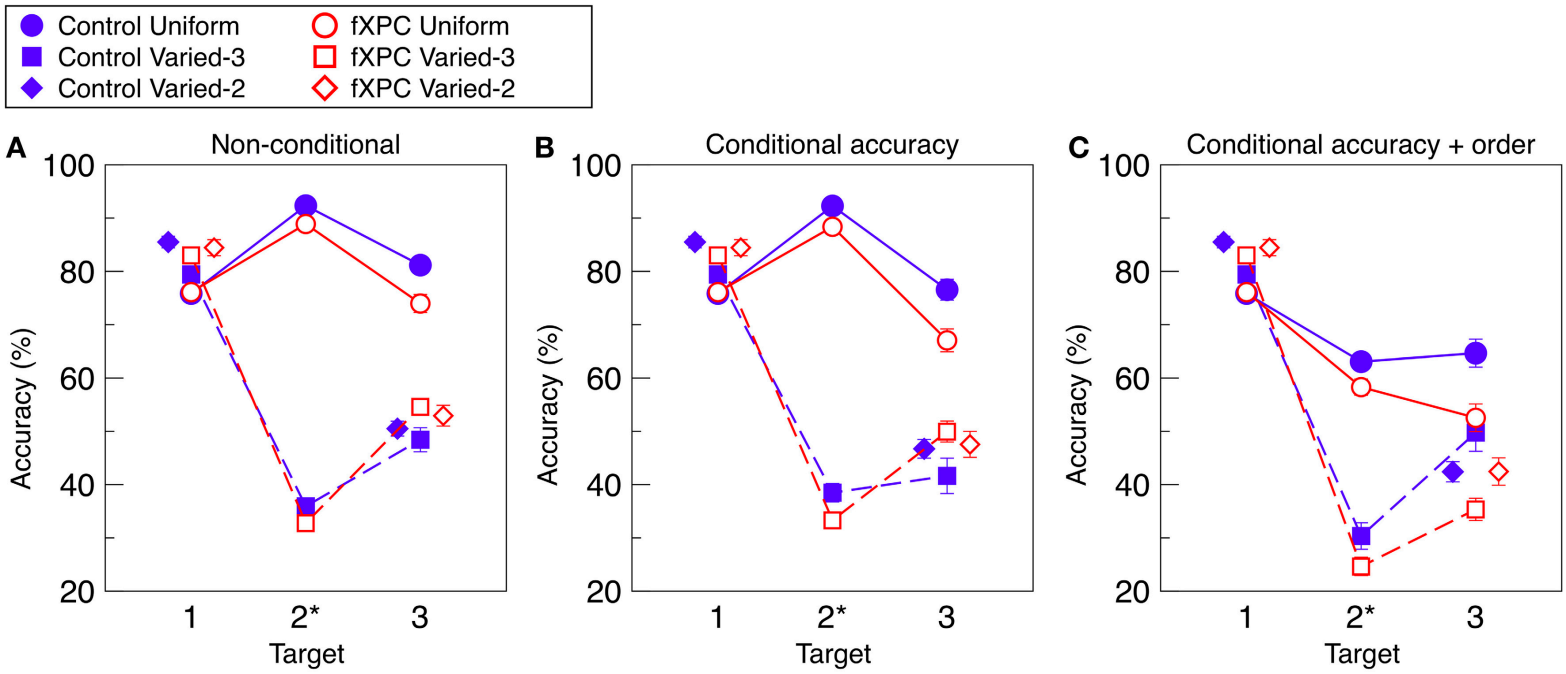
**Target identification accuracy. (A)** Non-conditional accuracy was computed as accuracy for each target regardless of accuracy for other items in the trial. **(B)** Conditional accuracy was computed for each target in which preceding target(s) were correctly identified. **(C)** Conditional accuracy was computed for each target in which preceding target(s) were correctly identified, and reported in the correct ordinal position. To prevent overlap, markers for the Varied–2 condition are horizontally offset. Target position 2 is marked with an asterisk because in the Varied-2 condition there are only two targets, one in position 1 and one in position 3. The AB is defined as lower accuracy for T3 than for T1 in a given condition. In **(A)** and **(B)** for both groups, and in **(C)** for controls, the AB was observed for the Varied–2 and Varied–3 conditions (all *p*s < 0.001), but not for the Uniform condition (*p*s < 0.17, 0.89, and 0.07, respectively). In **(C)** for fXPCs, the AB was observed in fXPCs for all three conditions (all *p*s < 0.001). Error bars represent standard error of the mean.

Figure 2A shows target identification accuracy for each target in each condition. Table 2 shows that the main effects of Condition and Target, as well as the Condition X Target interaction, were significant (all *p*s < 0.001). The main effect of Group was not significant (*F*_(1, 312)_ = 0.11, *p* = 0.74). The remaining interaction terms were not significant (all *p*s > 0.29). Controls demonstrated an AB in both Varied conditions (both *p*s < 0.001) but not in the Uniform condition (*t* = −1.40, *p* = 0.17). Likewise, fXPCs exhibited an AB in both Varied conditions (both *p*s < 0.001) but not in the Uniform condition (*t* = 0.50, *p* = 0.62).

**Table 2:** **ANOVA of Group X Condition X Target on accuracy**.

**Predictor**	**Accuracy**				**Conditional accuracy**				**Conditional accuracy + order**			
	**Df**	***F*-value**	***p*-value**	**η^2^_*p*_**	**Df**	***F*-value**	***p*-value**	**η^2^_*p*_**	**Df**	***F*-value**	***p*-value**	**η^2^_*p*_**
Group	1	0.11	0.74	0.00	1	0.35	0.56	0.00	1	0.76	0.38	0.00
Condition	2	22.35	<0.001	0.42	2	55.77	<0.001	0.32	2	11.91	<0.001	0.07
Target	2	140.80	<0.001	0.35	2	163.31	<0.001	0.35	2	12.32	<0.001	0.07
Group X Condition	2	1.19	0.31	0.01	2	1.64	0.20	0.01	2	0.42	0.66	0.00
Group X Target	2	0.87	0.42	0.00	2	0.77	0.38	0.00	2	4.85	0.01	0.03
Condition X Target	3	33.45	<0.001	0.44	3	33.11	<0.001	0.34	3	37.47	<0.001	0.27
Group X Condition X Target	3	1.26	0.29	0.01	3	3.66	0.06	0.01	3	1.75	0.16	0.02
Residuals	312				311				307			

*“Group” consisted of two factors: control or fXPC. “Condition” consisted of three factors: Uniform, Varied–2, or Varied–3. “Target” consisted of three factors: position 1–3, although the item in position 2 was not a target in the Varied–2 condition. A p < 0.05 was considered statistically significant*.

Di Lollo et al. (2005) observed in undergraduate students that T1 accuracy was higher in the Varied than Uniform condition (“T1 enhancement effect”). We examined this effect in controls, and observed this pattern in the Varied–2 (*t* = −2.85, *p* = 0.007) but not Varied–3 (*t* = −0.88, *p* = 0.38) relative to Uniform condition. Likewise, fXPCs demonstrated this effect in Varied–2 (*t* = −2.10, *p* = 0.04) but not Varied–3 (*t* = −1.77, *p* = 0.08) relative to Uniform condition.

#### 3.3.2. Conditional accuracy

Figure 2B shows target identification accuracy when preceding target(s) in that trial were accurately identified. Table 2 shows that the main effects of Condition and Target, as well as the Condition X Target interaction were significant (all *p*s < 0.001). The main effect of Group was not significant (*F*_(1, 312)_ = 0.35, *p* = 0.56). The remaining interaction terms were not significant (all *p*s > 0.20), although the Group X Condition X Target interaction trended toward significance (*F*_(3, 312)_ = 3.66, *p* = 0.057). Controls demonstrated an AB in both Varied conditions (both *p*s < 0.001) but not in the Uniform condition (*t* = −0.15, *p* = 0.89). Likewise, fXPCs exhibited an AB in both Varied conditions (both *p*s < 0.001) but not in the Uniform condition (*t* = 1.81, *p* = 0.08).

#### 3.3.3. Conditional accuracy + order

Figure 2C shows target identification accuracy when preceding target(s) in that trial were accurately identified and reported in the correct ordinal position. Table 2 shows that the main effects of Condition and Target, as well as the Group X Target and Condition X Target interaction were significant (all *p*s < 0.008). The main effect of Group was not significant (*F*_(1, 306)_ = 0.76, *p* = 0.38). The remaining interaction terms were not significant (all *p*s > 0.16). Controls demonstrated an AB in both Varied conditions (both *p*s < 0.002) but not in the Uniform condition (*t* = 1.91, *p* = 0.07). FXPCs exhibited an AB in all three conditions (all *p*s < 0.001).

#### 3.3.4. Summary of conditional analyses

In sum, requiring that preceding targets within a trial must have been correctly identified (“conditional accuracy”) or that targets must also be identified in the correct order (“conditional accuracy + order”) yields largely the same pattern of results as when these criteria were not applied. This suggests that if Di Lollo et al. (2005) had applied criteria necessary to interpret their data against the predictions of current models, their findings would have remained the same.

#### 3.3.5. Comparison of conditional and non-conditional analyses

**Figure 3:**
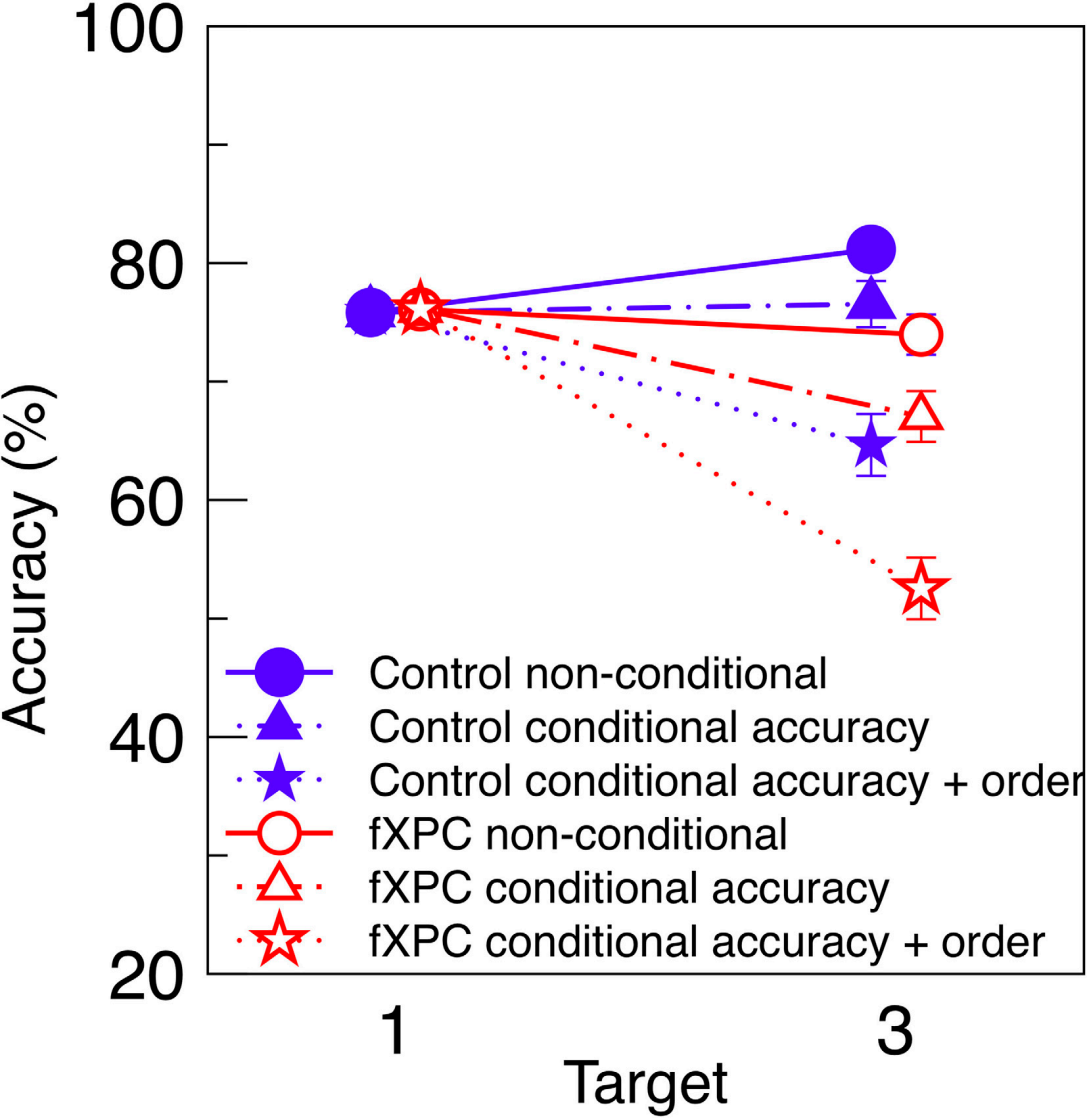
**Conditional and non-conditional accuracy**. Non-conditional accuracy (Figure 2A), conditional accuracy (Figure 2B), and conditional accuracy + order (Figure 2C) from the Uniform condition are re-plotted on the same axes. As expected, T3 accuracy decreases with increasing constraints on analysis. FXPCs were less accurate than controls (*F*_(1, 117)_ = 7.72, *p* = 0.006), and accuracy decreased with increasing analysis constraints (*F*_(2, 117)_ = 10.62, *p* < 0.001), while the Group X Contingency interaction was not significant (*F*_(2, 117)_ = 0.17, *p* = 0.85). The difference between conditional accuracy and conditional accuracy + order was significant for fXPCs (*t* = 2.16, *p* = 0.04) but not for controls (*t* = 1.82, *p* = 0.08). To prevent overlap, markers are horizontally offset. Error bars represent standard error of the mean.

To make our results more comparable to predictions by Dell'Acqua et al. (2009), Figure 3 displays conditional and non-conditional accuracy for the Uniform condition on the same axes. This is analogous to the third panel in Figure 3 of their manuscript. As expected, when T3 accuracy is computed from conditional accuracy + order, it is lower than when computed from conditional accuracy alone, and much lower than when computed from non-conditional accuracy. We tested whether the analysis contingency (non-conditional, conditional accuracy, or conditional accuracy and order) interacted with Group in a 3 × 2 ANOVA. We found that fXPCs were less accurate than controls (*F*_(1, 117)_ = 7.72, *p* = 0.006, η^2^_*p*_ = 0.06), and accuracy decreased with increasing analysis constraints (*F*_(2, 117)_ = 10.62, *p* < 0.001, η^2^_*p*_ = 0.15), while the Group X Contingency interaction was not significant (*F*_(2, 117)_ = 0.17, *p* = 0.85, η^2^_*p*_ = 0.00). The difference between conditional accuracy and conditional accuracy + order was significant for fXPCs (*t* = 2.16, *p* = 0.04) but not for controls (*t* = 1.82, *p* = 0.08).

### 3.4. Temporal ordering and memory

In Aim 2, we sought to more fully characterize the temporal dynamics of attentional selection. We addressed this by examining participant's perception of temporal order and level of inter-target competition.

#### 3.4.1. Perception of temporal order

If a participant correctly identified the targets and reported them in the correct order (e.g., reporting “T” before “C” in the example in Figure 1), we can be more confident that the participant perceived “T” as appearing earlier in time than “C.” Thus, from the trials where the targets were correctly identified, we calculated the percentage of trials in which the targets were reported in the correct order (“order accuracy”).

**Figure 4:**
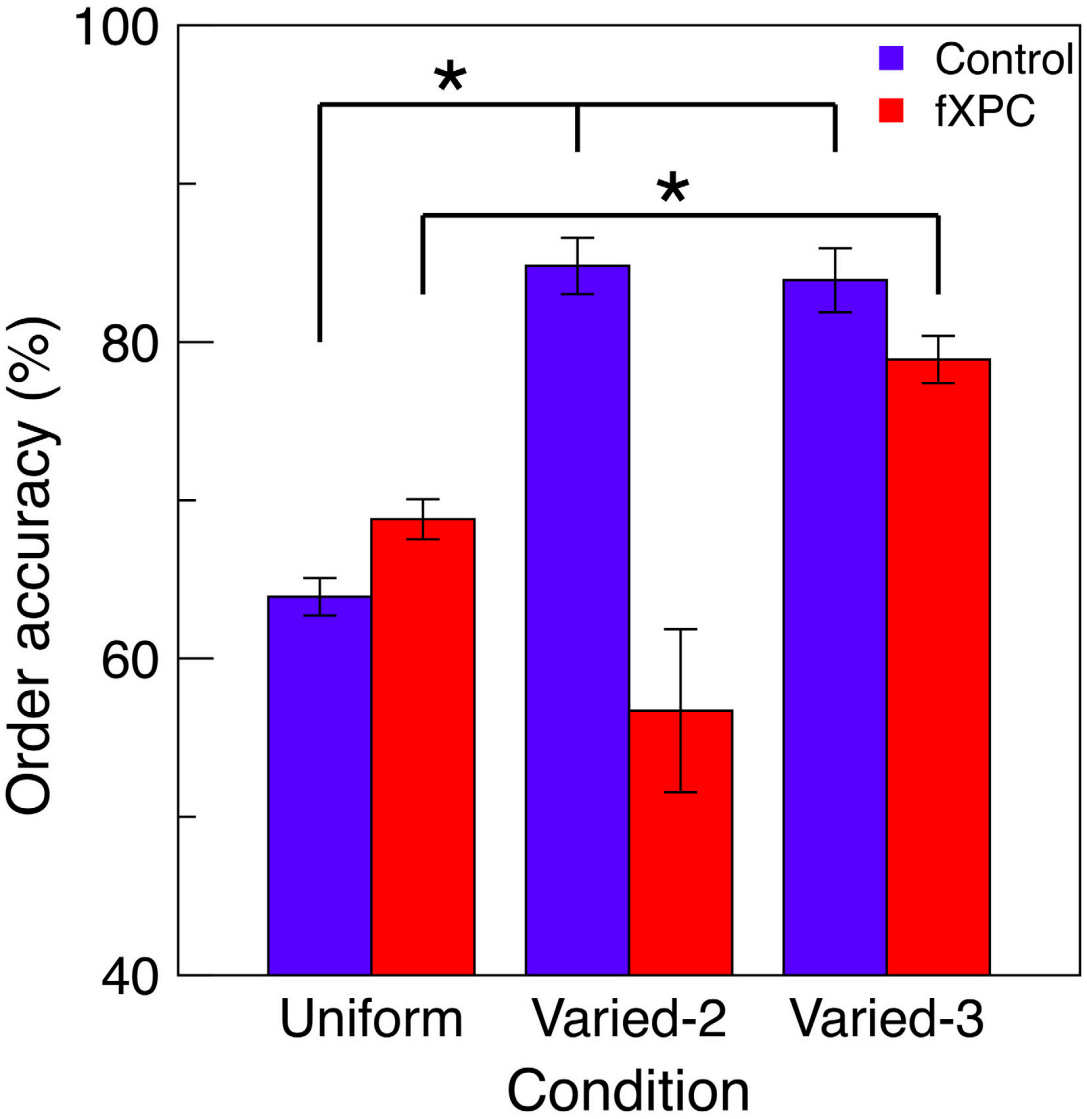
**Perception of temporal order**. For trials in which all targets were correctly identified, the order in which targets were reported was assessed for accuracy. Increased accuracy for order information represents improved perception of temporal order. In controls, order accuracy was higher in the Varied conditions than Uniform condition (both *p*s < 0.001), while the Varied conditions did not differ (*t* = 0.13, *p* = 0.89). In fXPCs, order accuracy was higher in the Varied-3 condition than Uniform condition (*t* = −2.54, *p* = 0.02), but did not differ between the other conditions (both *p*s > 0.20). However, the groups did not differ in any condition (all *p*s > 0.12). Error bars represent standard error of the mean. ^*^p < 0.05.

Figure 4 shows order accuracy for each condition, and Table 3 shows the ANOVA results. There was a main effect of Condition (*F*_(2, 115)_ = 5.83, *p* = 0.004) and Group X Condition interaction (*F*_(2, 92)_ = 5.07, *p* = 0.008), while the main effect of Group was not significant (*F*_(1, 115)_ = 1.76, *p* = 0.19). In controls, order accuracy was higher in the Varied conditions than Uniform condition (both *p*s < 0.001), while the Varied conditions did not differ (*t* = 0.13, *p* = 0.89). In fXPCs, order accuracy was higher in the Varied–3 condition than Uniform condition (*t* = −2.54, *p* = 0.02), but did not differ between the other conditions (both *p*s > 0.20). However, the groups did not differ within any condition (all *p*s > 0.12).

**Table 3:** **ANOVA of Group X Condition on order accuracy**.

**Predictor**	**Df**	***F*-value**	***p*-value**	**η^2^_*p*_**
Group	1	1.76	0.19	0.02
Condition	2	5.83	0.004	0.11
Group X Condition	2	5.07	0.01	0.10
Residuals	92			

*“Group” consisted of two factors: control or fXPC. “Condition” consisted of three factors: Uniform, Varied–2, or Varied–3. A p < 0.05 was considered statistically significant*.

#### 3.4.2. Inter-target competition

***3.4.2.1. T3 accuracy predicted by T1 accuracy***. Table 4 shows the ANOVA results. The main effects of Condition and T1 accuracy were both significant (both *p*s < 0.001), and Group X Condition interaction was significant (*F*_(2, 231)_ = 4.12, *p* = 0.02). In controls, T3 accuracy was higher when T1 was incorrect relative to correct, in both the Uniform (91.2% vs. 76.5%; *t* = 3.33, *p* = 0.002) and Varied–3 conditions (64.2% vs. 42.7%; *t* = 2.89, *p* = 0.006). Likewise, in fXPCs, T3 accuracy was higher when T1 was incorrect relative than correct, in both the Uniform (88.0% vs. 67.1%; *t* = 3.84, *p* < 0.001) and Varied–3 conditions (74.9% vs. 52.4%; *t* = 3.93, *p* < 0.001).

**Table 4:** **ANOVA of Group X Condition X paired target accuracy on current target accuracy**.

**Predictor**	**T3 accuracy**				**T1 accuracy**			
	**Df**	***F*-value**	***p*-value**	**η^2^_*p*_**	**Df**	***F*-value**	***p*-value**	**η^2^_*p*_**
Group	1	1.39	0.24	0.00	1	1.49	0.22	0.02
Condition	1	33.70	<0.001	0.26	1	1.92	0.15	0.00
Paired target accuracy	1	86.44	<0.001	0.23	1	57.22	<0.001	0.21
Group X Paired target accuracy	1	1.20	0.27	0.00	1	0.07	0.79	0.00
Condition X Paired target accuracy	1	1.23	0.29	0.01	1	0.19	0.83	0.00
Group X Condition	1	4.12	0.02	0.04	1	1.68	0.19	0.01
Group X Condition X Paired target accuracy	1	0.21	0.81	0.00	1	1.24	0.29	0.01
Residuals	154				156			

*“Group” consisted of two factors: control or fXPC. “Condition” consisted of three factors: Uniform, Varied–2, or Varied–3. A p < 0.05 was considered statistically significant*.

Although the Group X Condition interaction was significant (Figure 2A), the groups did not differ at any particular target or condition. For example, although T3 accuracy appears to be lower in fXPCs than controls in the Uniform condition, this difference is not significant (*t* = 1.49, *p* = 0.14). The interaction may be significant due to minor group differences across the conditions, such that fXPCs have slightly lower T3 accuracy than controls in the Uniform condition, but slightly higher T3 accuracy in the Varied–3 condition. No other main effects or interaction terms were significant.

***3.4.2.2. T1 accuracy predicted by T3 accuracy***. The main effect of T3 accuracy was significant (*F*_(1, 231)_ = 57.22, *p* < 0.001). In controls, T1 accuracy was higher when T3 was incorrect relative to correct, in both the Uniform (87.0% vs. 71.9%; *t* = 3.23, *p* = 0.003) and Varied–3 conditions (86.3% vs. 64.1%; *t* = 2.95, *p* = 0.007). Likewise, in fXPCs, T3 accuracy was higher when T1 was incorrect relative to correct, in both the Uniform (89.8% vs. 70.9%; *t* = 4.81, *p* < 0.001) and Varied–3 conditions (89.0% vs. 77.3%; *t* = 2.56, *p* = 0.01).

### 3.5. Relation to working memory tests

The mean digit span backward score did not differ between groups (controls = 8.4, fXPCs = 7.8; *t* = 0.69, *p* = 0.49), but fXPCs had significantly lower letter-number sequencing scores than controls (controls = 14.2, fXPCs = 11.9; *t* = 2.46, *p* = 0.02).

In Aim 3, we sought to determine whether the AB is better modeled as due to excessive or insufficient control. To address this, we tested whether executive WM (i.e., digit span backward or letter-number sequencing) predicted AB task performance, and whether this relationship differed between groups. We reasoned that if greater letter-number sequencing score (i.e., better executive WM) was associated with larger AB magnitude, this would provide evidence supporting the assertion that the AB is better modeled by excessive control. Meanwhile, the digit span backward task also requires updating and manipulation of information, but less so than in the letter-number sequencing task. Therefore, we did not expect that digit span backward score would be associated with AB magnitude. In Aim 4, we assessed whether the relationship between neuropsychological performance and AB magnitude differed between fXPCs and controls. Thus, we tested the interaction between Group and neuropsychological performance as predictors of AB magnitude.

Table 5 shows the linear regression results for both tests. First, we found that the main effects of Group (*F*_(1, 32)_ = 0.56, *p* = 0.46) and digit span backward score (*F*_(1, 32)_ = 3.45, *p* = 0.07), and the interaction of Group X score were all not significant (*F*_(1, 32)_ = 0.10, *p* = 0.75). Second, we found that the main effect of letter-number sequencing was significant, such that a greater score was associated with larger AB magnitude (*r* = 0.44, *p* = 0.007). The main effect of Group (*F*_(1, 32)_ = 0.64, *p* = 0.43) and the interaction of Group X score (*F*_(1, 32)_ = 0.82, *p* = 0.37) was not significant. In sum, we observed an association between AB magnitude and executive WM (i.e., letter-number sequencing) in the combined control and fXPC group. Letter-number sequencing score and AB magnitude were positively associated, supporting the interpretation that the AB is better modeled by excessive control than by insufficient control. FXPCs did not differ from controls in this regard. We did not observe an association between AB magnitude and digit span backward. We suggest that this could be due to decreased variability in digit span backwards scores relative to letter-number sequencing scores, which would decrease the likelihood that an association could be detected.

**Table 5:** **Linear regression of Group X neuropsychological test score**.

**Predictor**	**Digit span backward**				**Letter-number sequencing**			
	**Df**	***F*-value**	***p*-value**	**η^2^_*p*_**	**Df**	***F*-value**	***p*-value**	**η^2^_*p*_**
Group	1	0.56	0.46	0.11	1	0.64	0.43	0.10
Score	1	3.45	0.07	0.15	1	7.55	0.01	0.04
Group X Score	1	0.10	0.75	0.00	1	0.82	0.37	0.00
Residuals	33				33			

*“Group” consisted of two factors: control or fXPC. A p < 0.05 was considered statistically significant*.

### 3.6. Relation to ADHD symptoms

None of the ADHD sub-scale scores differed between groups on either the self- or observer-report (all *p*s > 0.12). No participants met ADHD criteria on both the self- and observer-report, though one control and three fXPCs met ADHD criteria on the Total Symptoms sub-scale of the observer and self-report, respectively. We reasoned that because ADHD symptoms and diagnosis prevalence tends to be increased in fXPCs, inattentive symptoms might impact performance on the AB task, and that this effect might be more pronounced in fXPCs. We used linear regression to examine the effect of Group X symptoms (either self-report or observer report) on AB magnitude. Neither main effects nor interactions were significant (all *p*s > 0.30).

### 3.7. Association with age and molecular variables

We tested the correlation between AB magnitude and molecular genetic measures in fXPCs. AB was not correlated with CGG repeat length (*r* = 0.08, *p* = 0.75) or with *FMR1* mRNA level (*r = −0.19*, *p* = 0.45). When we used the higher CGG repeat value for the participant who expressed two CGG values, the correlation with CGG repeat length remained not significant (*p* = 0.60).

## 4. Discussion

This study is the first to assess temporal attention in fXPCs using the AB task. Stimuli were presented foveally, so manipulation of attention instructions and distractor presence assessed the ability to ignore temporally intervening distractors. In this variant of the AB task, by manipulating attention demands (i.e., the type of items to be attended or ignored) and WM load (i.e., two or three items), we are able to explore the interaction between attention and WM in control participants and fXPCs. Our main finding is that fXPCs do not differ from controls in AB magnitude, indicating that the temporal dynamics of attentional selection are intact in fXPCs.

In Aim 1, we replicated the “spreading the sparing” effect, previously observed in undergraduate students, in a sample of adults aged 18–48. We also extended those findings by demonstrating that when applying progressively more stringent performance criteria, overall effects were maintained. This suggests that the results initially reported by Di Lollo et al. (2005) are robust. The additional performance criteria implemented in this study also aid interpretation of the original findings in light of models accounting for the AB, as will be described shortly. In Aim 2, we found that control participants were more likely to report targets in the correct order when there were only two letter targets (i.e., Varied–2 and Varied–3 conditions) than when there were three letter targets (i.e., Uniform condition). This suggests that with the switch from two to three targets, temporal information about the order in which each target appears is often lost. The likelihood of reporting targets in the correct order was higher in the Varied–3 condition than Uniform condition, suggesting that attention to an additional, intervening target (T2) is less detrimental when it belongs to a different stimulus set (i.e., T2 was a digit in the Varied–3 condition while T2 was a letter in the Uniform condition). In Aim 3, we found that in the combined control and fXPC group, increased letter-number sequencing score was associated with increased AB magnitude. Because letter-number sequencing requires executive WM, our results support the interpretation that the AB is better modeled by excessive control than by insufficient control. In Aim 4, we assessed whether fXPCs were impaired relative to controls in any of these measures.

We will first discuss implications of this study for understanding fXPCs. Second, we will briefly summarize competing models of attentional selection which make predictions about performance in this task. This will provide the background and theoretical framework required to understand the implications of this study. Third, we will discuss how our results replicate and extend previous findings. Fourth, we will discuss implications for models of the temporal dynamics of attentional selection. Finally, we will discuss implications for models of conceptualizing the AB as due to excessive or insufficient attentional control.

### 4.1. Implications for understanding neurocognitive function in fXPCs

In the combined control and fXPC group, letter-number sequencing performance was positively associated with AB magnitude. Both tasks require attending to letters and numbers, but the tasks differ in several ways. For example, they operate along different timescales of presentation. Attentional control is required to filter distracting information and can thus aid performance in the letter-number sequencing task. However, attentional control takes time to implement, so it can impair performance on tasks which require operation along very short timescales. This is what we observed in this study. When we examined groups separately, we found that fXPCs exhibited worse letter-number sequencing performance than controls, but did not differ in AB magnitude. This finding of a group difference in attention switching but not AB suggests that the temporal dynamics of attentional selection are intact in fXPCs. It also suggests that differential dynamics of attentional selection (i.e., in directing attention to targets or shifting attention from distractors) do not explain the WM impairments in fXPCs which have been reported previously (Brega et al., 2008; Grigsby et al., 2008; Cornish et al., 2009, 2011). Thus, impairments in WM might be due to other factors such as inability to filter distracting information, maintain information in memory, or manipulate information in memory.

Age did not affect performance in this task. We conclude this because when we included age as a covariate, the effect of age was not significant, and the pattern of results did not differ. This null result is interesting because FXTAS exhibits age-dependent penetrance (Jacquemont et al., 2004). There are several possible explanations for this null result. First, it might be the case that the effects of age-dependent penetrance are not observable until ages more advanced than in our sample. Our sample included adults aged 18–48, while prior studies in which an age effect was observed included adults aged 18–69 (Cornish et al., 2009) or only adults older than 50 (Jacquemont et al., 2004). Second, different symptoms might exhibit differential trajectories or levels of age dependence. For example, motor symptoms might manifest earlier in life and exhibit greater dependence on age while cognitive symptoms might manifest later in life and exhibit less dependence on age. Third, the effect of age might be less pronounced in fXPCs asymptomatic for FXTAS than in those with FXTAS.

To better understand the implications of differences in patterns of performance between fXPCs and controls, it would be helpful to understand the mechanisms that produce the AB. Therefore, in the next section we provide a brief summary of the theoretical models that have been proposed to describe and explain the AB and which have helped shape our understanding of attention and memory.

### 4.2. Models accounting for the AB

The AB refers to an impairment in perceiving the second of two targets presented closely in time in a RSVP stream (Raymond et al., 1992). With increasing time and number of intervening items, this effect diminishes. Thus, because the blink occurs only when two targets occur close together in time, the blink is thought to be due to difficulty engaging attention twice in a short time period (Nieuwenstein et al., 2009). In the current AB task, the AB was defined as decreased accuracy for T3 relative to T1.

The relevant models can be categorized as predicting that the AB is due to T1 processing or distractor processing (see Dux and Marois, 2009 for a review). Generally, the former models can be described as resource-depletion models, and the latter models can be described as distractor-based models. We will discuss examples of both types of models.

#### 4.2.1. Resource-depletion (RD) models

RD models stipulate that while T1 is being consolidated, T2 cannot be encoded and is susceptible to interference from trailing distractor stimuli (Ward et al., 1996). For example, according to interference theory, both targets enter limited-capacity WM and interfere with one another during retrieval (Shapiro and Raymond, 1994). According to bottleneck models, targets are rapidly identified during Stage 1 processing, but must then be consolidated into WM during capacity-limited Stage 2 processing (e.g., Chun and Potter, 1995). Similar to bottleneck models, the episodic simultaneous type/serial token (eSTST) model predicts that targets are identified during Stage 1, but must have identity and episodic information, such as the relative temporal position of the item in the stream, bound to a token during Stage 2 processing (Wyble et al., 2009). Unlike in the authors original STST model, the binding of items to separate tokens in the eSTST model allows for preservation of order information. These RD models predict that in this task, an AB should be observed in the Uniform as well as Varied conditions, because the same limited-capacity resources are devoted to T1 processing regardless of the presence of or attention to intervening distractors.

Distinct from other T1-based models, the boost and bounce model posits that WM capacity limitations play no role in the AB (Olivers and Meeter, 2008). Instead, target identification triggers an attentional boost, which is then followed by a bounce to prevent distractors from entering WM. Contiguous targets elicit a recurring boost, but after an intervening distractor appears, the bounce negatively affects identification of the trailing target.

#### 4.2.2. Distractor-based models

According to distractor-based models, the AB is produced by processes following the appearance of the distractor after T1. One such model is the TLC model, which we have already described (Di Lollo et al., 2005). As noted by Olivers et al. (2007), the TLC model thus assumes that resources are limited not at the low level of processing individual targets, but rather at a higher, executive level such that only one task can be prioritized at a time (i.e., monitoring or consolidation). This notion of limited executive level resources predicts that simultaneously performing two tasks which both require executive level resources should result in a decrement in performance, either in the non-prioritized task or both tasks. Indeed, the AB is reduced by manipulations that divide attention, such as the presentation of distracting visual motion and flicker (Arend et al., 2006), or the instruction to “pay a little less attention” (Olivers and Nieuwenhuis, 2006). These findings suggest that the AB is due to excessive attentional control. A related computational model posits that control processes suppress target detection while T1 is being consolidated, and would thus predict an AB in both the Uniform and Varied conditions (Taatgen et al., 2009).

#### 4.2.3. “Spreading the sparing”

One feature of AB task performance that is explicitly predicted by some models of the AB is the “spreading the sparing” effect, in which a trailing target presented in very close temporal proximity to the first target is often identified just as well as the first target (Olivers et al., 2007). Understanding the conditions under which sparing can be spread to additional targets and identifying capacity limits on the number of targets which can receive the spread would help inform and update these models.

### 4.3. Replicating and extending the “spreading the sparing” effect

In this study, we replicated in a sample of adults the “spreading the sparing” effect observed in an undergraduate sample (Di Lollo et al., 2005; Dell'Acqua et al., 2009). This sparing effect was observed as an AB in the Varied–2 and Varied–3 conditions, but not in the Uniform condition. When evaluating accuracy to trailing targets in an AB paradigm, it is critical to consider accuracy only from trials in which T1 performance was accurate (e.g., T3|T1+T2), in other words applying the *within-trial contingency (WTC) principle* (Dell'Acqua et al., 2009). We applied this principle in our “conditional accuracy” analysis and observed an overall similar pattern of results. While we expected conditional T3 accuracy to be lower than non-conditional T3 accuracy, a lack of marked difference suggests that T3 and T1 are often co-reported.

We then performed a more stringent “conditional accuracy + order” analysis. We reasoned that this additional constraint would allow us to visualize performance under conditions that best fit the assumptions of theoretical models. Specifically, these constraints ensure that T1 consolidation occurred successfully, and can therefore impact T3 perception or consolidation, and that episodic information about the temporal order in which the items appeared remained intact. Notably, neither RD models nor distractor-based models generate explicit predictions regarding the additional constraint of correct reporting of target order. Instead, this additional constraint reveals behavioral performance when 1) preceding targets were successfully encoded and 2) episodic information was correctly associated with each preceding target, such that relative temporal position of each target was maintained. Thus, this analysis imposes the most stringent criteria defining “correct task performance.” We observed an interactive Group X Condition effect on accuracy, such that fXPCs exhibited an AB in the Uniform as well as Varied conditions. In contrast, in the less stringent analyses fXPCs performed similarly to controls, exhibiting an AB in the Varied conditions but not the Uniform condition. Thus, this more stringent analysis revealed that fXPCs are subtly impaired relative to controls in terms of the ability to retain episodic information about temporal order.

### 4.4. Temporal ordering and memory

We examined the extent to which perception of temporal order is preserved in an AB task. This has been discussed previously to explain Lag 1 sparing (Hommel and Akyürek, 2005) and to identify whether the AB impairs perception of temporal order (Spalek et al., 2012). In the latter study, presence and absence of preceding and intervening distractors was systematically manipulated and found to have similar effects on the AB. Notably, the authors observed that distractors impaired perception of temporal order even while target identification accuracy remained unimpaired. According to their criteria, T2 and T3 could be reported in any order as long as (1) they were reported correctly and (2) T1 was reported correctly in the first ordinal position.

In the present study, to analyze perception of temporal order we required that T1 and T3 be: (1) correctly identified; (2) reported in the correct relative order; and (3) in the Uniform and Varied–3 conditions, that T2 was correctly identified. As described in the Materials and Methods, these constraints removed the potentially confounding effect of T2 stimulus type (i.e., either letter or number) on perception of temporal order. We found that in controls, order accuracy was higher in the Varied conditions than Uniform conditions. This was to be expected, because the Uniform condition, unlike the other conditions, contained an additional target from the same stimulus set as the other targets (i.e., letters), and episodic information for this target could be more easily lost or incorrectly attributed to another target. Additionally, we found that in fXPCs, order accuracy was higher in the Varied–3 condition than Uniform condition, but did not differ between the other conditions. The finding of higher order accuracy in the Varied–2 relative to Uniform condition in controls, but not fXPCs, suggests that fXPCs were attending to and encoding episodic information for the number distractor despite instructions to ignore all numbers.

To further examine the dynamics of attentional selection, we examined the effect of T1 accuracy on T3 accuracy, and the effect of T3 accuracy on T1 accuracy. In both instances, we found that accuracy for one target was higher when identification of the other target was incorrect. This supports models in which targets interfere with each other, regardless of which entered WM first (Shapiro and Raymond, 1994). However, it is less clear how other T1-based models would account for the effect of T3 accuracy on T1 accuracy.

No group differences in accuracy were observed, so we found little evidence for impaired temporal attention in adult male fXPCs asymptomatic for FXTAS relative to controls. Previous research reported that M pathway function was impaired in adult fXPCs (Kéeri and Benedek, 2009, 2012), and because the M pathway processes temporal information, this dysfunction might manifest as a disrupted AB. However, disrupting M pathway function in controls does not affect the AB (Nieuwenhuis et al., 2008). This supports models that describe the AB as the result of control processes, and suggests that the AB is more dependent on cortical than subcortical processing. Instead of a temporal processing impairment, we found that fXPCs exhibit relatively intact perception of temporal order, and similar patterns of inter-target competition in WM.

Kim et al. (2014) observed differences in neural processing of temporal WM in adult fXPCs, which is not necessarily inconsistent with our finding. For example, differential recruitment of brain regions in fXPCs relative to controls might nonetheless exhibit similar temporal dynamics, or result in similar response profiles. Alternatively, WM retrieval and attentional selection are related but distinct processes, such that one could be altered in fXPCs while the other is not. Exploring the relationship between brain activation and behavior, and clarifying which cognitive processes are impacted by *FMR1* mutations, is a potentially fruitful area for further research.

Overall performance was high, and effects were not modulated by ADHD symptoms, so it is unlikely that results are due to overall inattentiveness. This lack of relationship with attentiveness is consistent with previous findings that although alerting enhances target identification, alerting does not affect AB magnitude (Spalek and Lollo, 2010).

### 4.5. Excessive vs. insufficient control

To discern between different models of attentional selection that attribute the AB to either excessive or insufficient attentional control, we examined associations between the AB and traditional neurospsychological measures of executive WM. We reasoned that if the AB were due to excessive control, AB magnitude should correlate positively with letter-number sequencing score, but that if it were due to insufficient control, it should correlate negatively with letter-number sequencing score. Consistent with reported literature (Cornish et al., 2009, but see also Allen et al., 2011), we found that fXPCs exhibited lower letter-number sequencing scores than controls. We also found that both groups exhibited a positive association between letter-number sequencing score and AB magnitude, supporting models of the AB as the result of excessive control (Raymond et al., 1992; Olivers et al., 2007; Taatgen et al., 2009). These results are in line with findings that individuals who do not exhibit an AB effect (non-blinkers) are more efficient in ignoring distractors than blinkers (Martens and Valchev, 2009), and that blink magnitude increases when targets and distractors are more similar and therefore require more top-down control to distinguish (Chun and Potter, 1995).

Given lower letter-number sequencing scores in fXPCs, and a positive association between score and AB magnitude, we would predict that fXPCs also exhibit decreased AB magnitude (i.e., greater T3 accuracy), which was not the case. A potential confounding factor is that both the AB and letter-number sequencing tasks require a switch between attending to letters and numbers. It is possible that a cost associated with switching from T2 (number) to T3 (letter) identification resulted in decreased T3 accuracy, producing a larger rather than a smaller blink. Future studies using targets from a single stimulus set in a more traditional AB paradigm, or studies of executive WM that do not require task-switching, are needed to explore this possibility.

Finally, we should specify that our conclusion that the AB is better modeled by excessive rather than insufficient control is based on the finding of a correlation between AB magnitude and performance on the letter-number sequencing task, which requires executive WM. The idea that these two tasks might be related is not new; the relationship between AB duration and letter-number sequencing performance has been assessed previously (Gillard-Crewther et al., 2007). Executive function is required to switch between letters and numbers in the letter-number sequencing task, which puts higher load on the central executive of WM, and is similarly required to switch between letters and numbers in the AB task. However, because successful letter-number sequencing performance involves memory for increasingly longer lists of items, while this AB task requires memory for only 2-3 items, the tasks are not completely analogous. Future studies utilizing direct measures of attentional control in conjunction with the AB task will help clarify this emerging evidence for the AB as a task of excessive attentional control.

### 4.6. Limitations

One limitation of our study is our choice of AB task. MacLean and Arnell (2012) argue that the AB cannot be properly assessed without sampling at least two trailing target lags: one shorter lag within the window of a typical AB, and one longer lag at which point the AB has typically resolved. Otherwise, any group effects could be due to differences in T2 accuracy alone. Our task design, which held trailing target lag constant, allowed us to examine the effect of attend instructions and intervening distractors. Instead of using accuracy from a long inter-target lag condition as a control condition, we used accuracy from the Uniform condition. Although this design best suits the goals of this study, differences between our task design and traditional AB task designs limit the extent to which our findings can directly extend existing literature.

Another limitation of this study is that participants were allowed to identify targets in any order. This replicates how the task was administered in previous studies, so that identification accuracy was the primary outcome measure. However, because participants were not explicitly required to encode order information, different response strategies may have caused variations in the order of responses and affected our analyses of temporal perception. We speculate that a requirement to encode order information would make the task more challenging and potentially more sensitive to group differences. Thus, future studies manipulating attention to temporal information are needed for a more complete understanding of the temporal dynamics of attention in fXPCs.

A third limitation of this study is that in the conditional accuracy and conditional accuracy + order analyses, by becoming more stringent with the selection criteria, fewer and fewer trials for T3 were eligible for analysis. Thus, one must consider the difference in the number of included trials when comparing the results of these analyses. Similarly, the number of included subjects could differ (e.g., if one participant consistently reported targets in the incorrect order).

A fourth limitation of this study is that we did not include female fXPCs. We reasoned that male fXPCs, because they lack a second, unaffected *FMR1* allele and are at greater risk of developing FXTAS, would be more likely than female fXPCs to exhibit group differences in cognitive performance. However, reports of impairments in female fXPCs (Goodrich-Hunsaker et al., 2011a,b) but not male fXPCs in the same task (Wong et al., 2012), or of enhanced psychomotor speed in female fXPCs but not male fXPCs (Goodrich-Hunsaker et al., 2011c; Wong et al., 2012) challenge this potentially overly simplistic view.

Finally, although we examined processes that may be affected in FXTAS, the fXPCs we studied were asymptomatic for FXTAS. To identify whether cognitive impairments precede or characterize FXTAS, longitudinal studies are needed. We observed no differences in temporal attention between fXPCs and controls, but this could be because participants in our sample will not go on to develop FXTAS, temporal attention is truly unaffected in fXPCs, our sample size was insufficient to detect a small effect, or the AB task was insensitive to the specifically affected processes.

### 4.7. Conclusion

This study was the first to examine the dynamics of temporal attention in fXPCs. We found no differences between adult male fXPCs asymptomatic for FXTAS and controls, suggesting that attentional selection processes were intact. Meanwhile, fXPCs exhibited impaired attentional control, observed as impaired performance in the letter-number sequencing task. Understanding what cognitive processes are intact or impaired in fXPCs may facilitate early identification of individuals most at risk for developing FXTAS. Furthermore, we replicated and extended findings in controls using an AB paradigm. Results from this study support models of attentional selection that posit that the AB is due to excessive, and not insufficient, attentional control. However, future studies are needed to develop a more complete understanding of the dynamics of temporal attention and the effect of control processes on those dynamics.

## Author contributions

Ling M. Wong played a primary role in experiment design, data analysis, data interpretation, and manuscript preparation. FT performed the molecular genetics analysis. Susan M. Rivera participated in the design of the study, and supported data interpretation and manuscript preparation. Tony J. Simon participated in the design of the study, and supported data interpretation and manuscript preparation. All authors read and approved the final manuscript.

## Funding

This work was supported by National Institute of Health (NIH) grants: NIA RL1 AG032119, NINDS RL1 NS062412, NIDA TL1 DA024854, and NIMH MH078041. This work was also made possible by a Roadmap Initiative grant (UL1 DE019583) from the National Institute of Dental and Craniofacial Research (NIDCR) in support of the NeuroTherapeutics Research Institute (NTRI) consortium. The NIH had no further role in study design; in the collection, analysis and interpretation of data; in the writing of the report; and in the decision to submit the study for publication.

### Conflict of interest statement

The Review Editor Dr. Claudine Kraan declares that, despite having collaborated with one of the authors, the review process was handled objectively and no conflict of interest exists. The authors declare that the research was conducted in the absence of any commercial or financial relationships that could be construed as a potential conflict of interest.

## Abbreviations

AB: attentional blink
CAARS: Conners' Adult ADHD Rating Scale
FMRP: fragile X mental retardation protein
fXPC: fragile x premutation carrier
FSIQ: full scale IQ
FXS: fragile X syndrome
FXTAS: fragile x-associated tremor/ataxia syndrome
LNS: letter-number sequencing
TLC: temporary loss of control
RD: resource-depletion.
